# Free-Living Dietary Intake in Tactical Personnel and Implications for Nutrition Practice: A Systematic Review

**DOI:** 10.3390/nu13103502

**Published:** 2021-10-03

**Authors:** Kristen L. MacKenzie-Shalders, Angela V. Tsoi, Ka Wing Lee, Charlene Wright, Gregory R. Cox, Robin M. Orr

**Affiliations:** 1 Nutrition and Dietetics, Faculty of Health Sciences and Medicine, Bond University, Robina 4226, Australia; angela.tsoi@student.bond.edu.au (A.V.T.); kawing.lee@student.bond.edu.au (K.W.L.); charlene.wright@griffithuni.edu.au (C.W.); gcox@bond.edu.au (G.R.C.); 2 School of Medicine and Dentistry, Centre of Applied Health Economics and Menzies Health Institute Queensland, Griffith University, Gold Coast 4222, Australia; 3 Tactical Research Unit, Bond University, Robina 4226, Australia; rorr@bond.edu.au

**Keywords:** diet, nutrition, health, performance, occupation, police, fire, military

## Abstract

Tactical personnel (including military, law enforcement, and fire and rescue) are responsible for ensuring national and public safety. Dietary intake is an important consideration to support optimal health and performance. The aims of this systematic review were to: (1) describe the reported free-living dietary intake (energy and macronutrients) of tactical personnel, and (2) describe the practical implications of reported dietary intakes to support the physical and dietary requirements of tactical personnel. A systematic search of databases (MEDLINE, EMBASE, CINAHL and Web of Science) was conducted following the PRISMA guidelines. English and full text research articles were identified and screened against inclusion and exclusion criteria. Demographic and dietary intake data were extracted, tabulated, and synthesized narratively. The quality of the studies was assessed using the Academy of Nutrition and Dietetics Quality Criteria Checklist. Twenty-two studies (15 military, 4 law enforcement, and 2 fire and rescue) were eligible to inform this review. The volume of evidence suggested that tactical personnel met dietary protein and exceeded dietary fat recommendations but failed to meet energy and carbohydrate recommendations. Therefore, practical approaches to support optimized energy, fat and carbohydrate intake in tactical personnel is important.

## 1. Introduction

Optimizing dietary recommendations, and subsequent intake, to support health, well-being and performance is important for tactical personnel [1]. Tactical occupations, including military, law enforcement, and fire and rescue emergency services (SES) [2], have many important dietary considerations and challenges. Several studies have reported sedentary behaviors, chronic disease risk, and obesity rates similar to, or higher than, those seen at a population level [3,4,5,6,7,8,9,10,11]. However, tactical occupations present a range of shared and unique challenges that can impact dietary intake [12]. Physically and mentally demanding occupational tasks [13]; heightened periods of physical exertion [14]; over-time, shift work and lack of structured breaks; working in extreme conditions (extreme heat, altitude, and low humidity) [15,16]; and specific occupational risks serve as examples [17,18]. Many of these factors, including long working hours, shift work, and job strain (in males) have been associated with poor diet quality [19], in turn having implications for the health, well-being, and performance capability of tactical occupations. 

While a focus on nutrition strategies that promote good health are important, and population-specific recommendations can be applied, dietary recommendations to optimize macronutrient intake, and general nutrition, for performance are also warranted [20]. Tactical personnel should also maintain a requisite level of physical fitness to perform job tasks optimally and therefore sometimes sports nutrition recommendation are also applied [21,22]. Tailored dietary recommendations that provide an appropriate energy and macronutrient intake will support physical fitness [23] and likely optimize the performance of tactical personnel. A recent cross-sectional study reported that law enforcement personnel place high importance on consuming nutritious food, food high in vitamins and minerals, and that have a high protein content [20]. To the authors knowledge, several other studies have documented tactical personnel’s’ free-living dietary intake [15,19,24,25,26,27]. However, synthesizing current evidence to inform macronutrient requirements and broader nutrition strategies for tactical occupations would be beneficial.

Several studies have previously demonstrated that healthy eating interventions can positively impact dietary intake and behaviors in tactical personnel [14,28,29]. Therefore, to best support valid nutrition strategies for tactical occupations, documenting and interpreting their dietary intake and contextualizing their intake in consideration of their dietary requirements is a required initial step to inform recommendations. In the public or scientific domain, there are no comprehensive, up-to-date dietary guidelines specific for tactical occupations. While there are military dietary reference intakes (MDRIs) which are based on the recommended daily allowances (RDAs)—for military personnel which are the nutrient standards intended to meet the requirements of majority of healthy Americans aged ≥2 years [30,31]—the MDRIs are approximately 20 years old and the RDAs have since been updated.

There is no known systematic review exploring dietary intake specific for tactical occupations. Therefore, the aims of this systematic review were to: (1) describe the reported free-living dietary intake (energy and macronutrients) of tactical personnel including military, law enforcement, and fire and rescue, and (2) describe the practical implications of reported dietary intakes to support the physical and dietary requirements of tactical personnel. This research will assist in understanding tactical personnel’s free-living dietary intake and, through comparison to other available information (e.g., population normative data), support the development of evidence-based dietary interventions and recommendations.

## 2. Materials and Methods

This systematic review was conducted and reported following the Preferred Reporting Items for Systematic Reviews and Meta-analyses (PRISMA) protocol [32]. The protocol was prospectively registered with the International Prospective Register of Systematic Reviews (PROSPERO registration: CRD42021224080).

### 2.1. Search Strategy

Four electronic databases were searched, including Medline (via PubMed), EMBASE (via Ovid), Cumulative Index to Nursing and Allied Health Literature (CINAHL via EBSCOhost) and Web of Science. Publications were restricted to those published from 1990 up until 28 October 2020. No language restrictions were applied. A combination of keywords designed for PubMed were used and converted for other databases using CREBP-SRA Polyglot Search Translator [33]. For each database, converted keywords were checked for accuracy and modified as required. The entire search strategy can be seen in Table S1. Additional relevant studies were identified through forward and backward (‘snowballing’) citation searching [34].

### 2.2. Eligibility Criteria

To be eligible for inclusion, each study had to satisfy criteria regarding population, intervention, comparison, outcome, and study design (Table 1). Published studies involving tactical personnel (including military personnel, law enforcement personnel, fire and rescue personnel, and active-services personnel), aged ≥18 years, and following a free-living diet (e.g., with no restrictions and no food provided) were included. Studies that failed to report on any outcomes of interest. were excluded as were studies with interventions centred on dietary supplementation of macronutrients or diets for special training or altitude, as these impact dietary requirements and may not reflect usual free-living macronutrient intake.

### 2.3. Study Screening

Duplicate records identified during the search strategy were removed using Endnote (version 9) [35]. Initially, records were identified and marked as ineligible and excluded via text-mining in Endnote by identifying keywords not relevant to this review (e.g., children and animal studies) [36]. Two authors (AT and KL) conducted the title and abstract screening for eligibility in Covidence a web-based, review management, software platform [37], with conflict resolution was completed by a third author (KMS). The full texts of potentially eligible papers were reviewed to confirm eligibility by two independent investigators (AT and KL). Disagreements were managed by consensus or a third reviewer when required (KMS). One publication in a language other than English was transcribed using Google Translate^®^ before the full-text screening [38].

### 2.4. Data Extraction

The primary outcome of this review was dietary intake including energy, carbohydrate, protein, and fat. Data from the included studies were extracted into an electronic spreadsheet by two authors (AT and CW) and checked for accuracy by two separate authors (KMS and KL). Items extracted included study details (author, publication date, country, study design, setting), participant characteristics (age, sex, height, weight, body mass index), and key methodologies and assessment tools. Data were extracted for baseline, pre-intervention, or pre-deployment data. If a study did not report body mass index yet height and weight data was available, BMI was calculated using the formula BMI = kg/m^2^ where kg is a person’s weight in kilograms and m^2^ is their height in meters squared. Dietary data were converted to the same units used in the military dietary reference intakes (MDRI) for comparison. Dietary intake was reported in kcal, protein in grams, and carbohydrate and fat intake as percentage of energy intake. As required, kilojoules were converted to kcal and grams or kcal were converted to % total energy. If studies stratified participants, the Cochrane formula for ‘combined groups of means and standard deviations into a single group’ was used [39]. The analytical approach used narrative description that reported dietary intake of energy, carbohydrate, protein and fat, occupation of participants, and outcomes of the study.

### 2.5. The Military Dietary Reference Intakes (MDRIs)

The reported dietary intake of energy, carbohydrate, protein, and fat in included studies were compared to the military dietary reference intakes (MDRIs) [31]. The reference intakes were adapted from the sex- and age-specific recommended daily allowances (RDAs). The RDAs are the nutrient standards intended to meet the requirements of majority of healthy Americans aged ≥2 years [40]. While these guidelines may not be applicable to some international tactical personnel, these were the only broadly applicable guidelines in international scholarly literature. 

### 2.6. Quality Assessment

The Academy of Nutrition and Dietetics Quality Criteria Checklist (QCC) for primary research from the Academy of Nutrition and Dietetics Evidence Analysis Manual [41], was used to assess study quality. The QCC includes ten validity questions based on the Agency for Healthcare Research and Quality domains for research studies. It evaluates whether studies addressed selection, bias, generalizability, data collection, and analysis to a sufficient standard in their reporting. Occasionally, a major question is not applicable (N/A) to a specific study. For example, the use of N/A is indicated in validity question 3 of the checklist which looks at “were study groups comparable or was an appropriate reference standard used” when only one group was studied, with no comparison group. Two investigators independently completed the assessment of study quality (AT and KL). Disagreements were managed by consensus. Studies were given an overall rating as “positive”, “negative” or “neutral”. No studies were excluded based on the overall quality rating.

## 3. Results

### 3.1. Search Results

**Figure 1:**
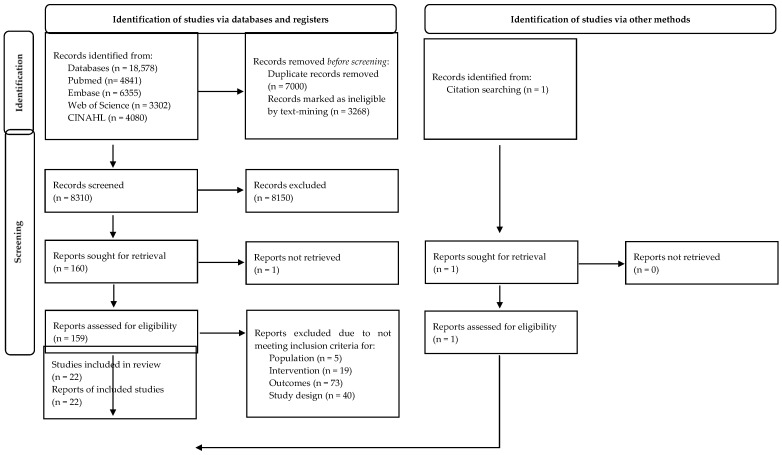
Preferred Reporting Items for Systematic Reviews and Meta-Analysis flowchart of the search results and the included studies.

A total of 18,578 records were identified through database searches (Figure 1). After duplicates were removed, 8310 records remained for title and abstract screening. Overall, 158 studies were identified by the primary search and one study through forward/backward citation searching resulting in 159 studies being reviewed in full text. Of these studies an additional 137 were excluded due to the population (n = 5), intervention (n = 19), outcomes (n = 73), and study design (n = 40) not meeting inclusion criteria. A total of 22 studies were included for this review.

### 3.2. Study and Sample Characteristics

Characteristics of included studies can be seen in Table 2. All included studies were published between 1990 and 2020. They involved military personnel (n = 16) [42,43,44,45,46,47,48,49,50,51,52,53,54,55,56,57], law enforcement personnel (n = 4) [26,58,59,60], or firefighters (n = 2) [61,62], and were from a range of countries, including the United States of America (USA) (n = 9) [26,42,43,44,50,51,52,54,62], Israel (n = 3) [46,47,57], the United Kingdom (UK) (n = 2) [53,59], Italy [55], Belgium [48], Brazil [58], Canada [60], Cameroon [49], Thailand [45], Iran [56], and Australia [61] (n = 1). 

Most studies reported both male and female data [26,46,47,49,50,51,54,59,60,61], eight included male data only [42,45,48,55,56,57,58,62], and three did not specify sex [43,44,53]. Sample sizes ranged from 19 to 4078 participants. The mean age of participants in the included studies ranged from 19 (±1) to 43 (±7) years (Table 2). BMI was reported in 16 studies [45,46,47,50,51,52,53,54,55,56,57,58,59,60,61,62], was able to be calculated in three studies [42,43,44], and was not reported or able to be calculated in three studies [26,48,49]. Available BMI data for females ranged from 23 (±3) to 26 (±5) kg/m^2^ and males ranged from 22 (±4) to 30 (±3) kg/m^2^ (Table 2).

Methods for dietary assessment used in the included studies include food frequency checklist or questionnaire [42,44,46,47,48,49,50,51,55,56,62], 24 h recall [42,54,57,58,61], 3-day food record [43,45], 4-day food record [48,53], 7-day food record [26,59], various nutrition and lifestyle questionnaires [49,52,54], and photographs of food [58,60]. 

Almost one third of included studies did not compare dietary intakes with a dietary reference guideline (n = 7) [43,48,50,55,58,59,61]. Of those that did, four strictly used the MDRIs [47,52,53,57] Two studies used a combination of MDRI with sports nutrition guidelines [54], and Dietary guidelines for Americans (DRAs) [44]. Alternate reference guidelines included Institute of Medicines dietary reference intakes (DRIs) (n = 4) [46,51,60,62], U.S. Dietary Goals (n = 2) [26,42], Department of agriculture’s dietary guidelines (n = 2) [49,51], International Network of Food Data Systems [49], Alternative Healthy Eating Index-2010 [56], Dietary Reference Intake for Thais [45], Military recommended dietary allowances (MRDA), and Safe and adequate daily dietary intakes (ESADDI) according to military Nutrition Allowances, Standards, and Education (n = 1) [42].

### 3.3. Reported Dietary Intakes

All included studies assessed the energy intake of participants. Regardless of sex, all participants were below the MDRI for total energy intake (Table 3). 

The majority of studies, except for two [49,58], assessed protein intake (n = 20, 91%). Eleven studies, involving military personnel, reported male specific results with the majority (n = 8, 73%) exceeding the MDRI of 91 g/day [42,46,48,52,54,55,57]. Three studies reported participants consuming <91 g/day (n = 3, 27%) [45,47,50]. One study involving law enforcement personnel reported males mostly meeting the MDRI with 90 g/day [59] and one study involving firefighters reported males exceeding the MDRI with 123 g/day [62]. Five studies involving military personnel reported female specific results with the majority exceeding the MDRI of 72 g/day (n = 4, 80%) [46,47,52,54], and one study consuming <72 g/day [50]. One study involving law enforcement personnel reported females mostly meeting the MDRI with 71 g/day (99% of the MDRI) [59]. 

The majority of studies assessed carbohydrate intake (n = 20, 91%) except for two [49,58]. Eleven studies, involving military personnel, reported male specific results with the majority (n = 10, 91%) finding participants below the MDRI of ≥55% of total energy [42,45,46,47,48,50,52,54,56,57], and one study reported males meeting the MDRI with 56% of total energy from carbohydrates [55]. One study involving law enforcement personnel [59], and one involving firefighters [62], reported males were below the MDRI for carbohydrates. Five studies involving military personnel reported female specific results and they were all below the MDRI of ≥55% of total energy [46,47,50,52,54]. As was the one study with female law enforcement personnel [59].

All but one study [49], assessed fat intake (n = 21, 95%). Eleven studies, involving military personnel, reported male specific results with the majority (n = 8, 73%) exceeding the MDRI of ≤30% of total energy [42,46,47,48,49,50,52,54,57], with the remaining three studies meeting the MDRI (n = 3, 27%) [45,55,56]. Two studies involved male law enforcement personnel [58,59], and one study involved male firefighters [62], and these studies likewise reported results above the MDRI. Five studies, involving military personnel, reported female specific results with the majority (n = 4, 80%) exceeding the MDRI of ≤30% of total energy [46,47,50,52], and one study below the MDRI with 29% of total energy from fat. The one study with female law enforcement personnel reported participants exceeding the MDRI [59]. 

### 3.4. Quality Assessment

Of the 22 studies, 16 were rated as positive and six were rated as neutral as per the Academy of Nutrition and Dietetics Quality Criteria Checklist. The critical appraisal results can be seen in Table S2. Of the studies that were graded neutral the most common domain impacting the studies’ scores were their lack of describing the handling of withdrawals, followed by reported dietary intake cannot be comparable due to not presenting means and standard deviations, their statistical analysis was not mentioned in detail, and/or their limitations were not discuss in detail. For Nkondjock et al. [49], reported energy and macronutrient intakes were stratified across three different personnel including officers, warrant officers, and enlisted men however, tabulated participant numbers were inconsistent with those reported in the body of text. 

## 4. Discussion

This systematic review aimed to: (1) describe the reported free-living dietary intake (energy and macronutrients) of tactical personnel including military, law enforcement, and fire and rescue, and (2) describe the practical implications of reported dietary intakes to support the physical and dietary requirements of tactical personnel. The volume of evidence from the reported studies suggest that, comparatively to the MDRI, tactical personnel met dietary protein and exceeded dietary fat recommendations but failed to meet energy and carbohydrate recommendations. Similarly, a recent systematic review conducted in athletes who like some tactical personnel perform regular training, reported that team-sport athletes met or exceeded recommendations for protein and/or fat but did not meet energy and carbohydrate recommendations [63]. This, despite these athletes having access to high-quality research studies and up-to-date consensus dietary guidelines [63,64,65].

### 4.1. Suboptimal Free-Living Energy and Carbohydrate Intake

In comparison to the MDRI recommendations, free-living dietary energy and carbohydrate intake across military personnel, law enforcement personnel, and firefighters, failed to reach recommended values, regardless of sex. An appropriate dietary energy intake is important for tactical populations as their occupational activities can be physically demanding [66,67,68]. Inadequate energy intake can lead to weight loss, decreases in lean muscle mass, and decreases in bone density, which can affect daily performance, increase injury risk, and prolong recovery time [69]. Tactical occupations are renowned for phases of energy deficiency and recovery, and a potential risk for Relative Energy Deficiency in Sport (RED-S) syndrome in military personnel has previously been reported [69]. Therefore, while the reported BMI of tactical personnel did not support a chronic energy deficiency as they were all >25 kg/m^2^ (e.g., categorized as normal through to obese), it is unclear whether higher BMI values are reflective of increased lean (muscle) mass or fat mass. Some tactical occupations personnel exhibit higher levels of physical activity and/or strength training while others may intermittently be active or be relatively sedentary [70,71]. Studies describing the physical activity levels of tactical personnel can help advise specific dietary recommendations [72]. Several studies have documented tactical personnel’s physical fitness is associated with their diet and nutrition status [73,74,75]. Of course suboptimal energy intakes may reflect under-reporting, despite this, it is unclear whether suboptimal energy intakes may be reported by some tactical personnel due to a culture surrounding lean body composition goals and/or restricted dietary intake in these occupations. Previous studies have shown these trends in athletes [76,77], and we have recently reported high uptakes of special diets in law enforcement personnel including low carbohydrate, low fat, and other potentially restrictive diets [20], which may extend to other tactical occupations. 

A systematic review of shift workers’ dietary intake has previously described unconventional meal patterns, skipping meals, and consuming more food at ‘unconventional’ times [78]. For example, it is common in shift workers to see periods of fasting followed by excessive dietary intake (e.g., through the use of convenience or take-away foods, that are high in energy, fat and sugar) [78]. Copp et al., found military personnel skipped breakfast [42], and Rahmani et al., reported an inverse association between healthy eating and odds of depression and anxiety [56]. Based on the available evidence, while overall energy and carbohydrate intake appears to be suboptimal, further exploration on the free-living type and timing of dietary energy and carbohydrate intake in tactical occupations is warranted [2].

### 4.2. Excessive Free-Living Dietary Protein and Fat Intake

Tactical personnel reported an adequate amount of dietary protein in comparison to the MDRI guidelines. Dietary protein intake supports a range of anabolic physiological functions including the synthesis of muscle proteins, hormones, enzymes, and antibodies. Adequate intake of protein can optimize occupational performance and insufficient protein intake can lead to protein catabolism, skeletal muscle weakness or wasting, illness, injuries, and longer recovery time [64,79]. 

Studies have repeatedly demonstrated that protein intake is adequate in the general population with some exceptions, (e.g., vegetarian, vegan and older populations [80,81,82]). However, the MDRI reference range is based around population recommendations (e.g., 0.8–1.5 g per kg body weight) but incorporates a higher upper level for enhanced physical activity or muscle mass accretion [31]. In addition, some studies have demonstrated lower protein intakes in night shift workers, alongside higher levels of snacking, so dietary adequacy may be impacted by work schedules and rostering [78]. While this systematic review has reported protein intakes that meet recommendations, it is plausible that targeted, quality protein intakes may be beneficial where there is low food availability to support nutrient intake and satiety, or for individuals undergoing regular resistance exercise [64,83]. 

Due to its popularity and potential benefits, dietary protein is commonly supplemented [84]. While only two of the studies specifically reported that participants were using protein or carbohydrate supplement food items (such as protein bars and protein powders [41,50]), it is possible that the use of these products is higher with a previous study reporting that approximately 40% of law enforcement personnel use protein supplements [20]. As protein supplements can be costly, can displace other foods and nutrients, and can potentially include other contaminants that may have side effects (e.g., added caffeine impacting alertness and sleep cycles), it is important that supplementation is considered within future dietary guidelines [64,84]. 

The majority of included studies reported fat intakes above the MDRI suggestion of being less than 30% of total energy intake. For the studies that were within this recommendation, the reported dietary fat intake was at the upper end of the recommended range. Excessive fat, particularly saturated fat, intake can cause health issues such as high LDL-cholesterol levels, increase cardiovascular disease (CVD) risk, and weight gain in tactical personnel [39]. This finding is of concern given the higher levels of obesity and BMI that has been reported in police [85], firefighters [86], and military personnel [87], when compared to the general population, and the association of these characteristics to CVD risk [88]. However, over two thirds of included studies (n = 15, 68%) did not report saturated fat separately. Dietary patterns and behaviors that may contribute to higher fat intakes amongst tactical personnel include higher levels of snacking, high use of take-away foods, or a lower intake of fiber, fruit, or vegetables [20,78]. Similar to other active populations, tactical personnel may benefit from the inclusion of mono- and poly-unsaturated fat-based foods (e.g., fish, nuts, and seeds) to help meet energy requirements and provide anti-inflammatory benefits [31,63]. 

### 4.3. Practical Implications and Priority Areas for Intervention 

Based on the volume of evidence presented in this review, priority areas for intervention programs and nutritional guidelines in tactical occupations should support (1) an adequate dietary energy and carbohydrate intake; (2) controlled dietary fat intake with consideration of population recommendations of reducing saturated fat and prioritizing unsaturated fat intake; and (3) moderate, high-quality protein intake that is not prioritized at the expense of other macronutrients. Where possible, nutrition professionals should interpret these recommendations to ensure they are food-based and are interpreted with consideration of tactical personnel’s varied occupational demands. In addition, the consideration of other dietary factors including barriers to dietary intake and their solutions (e.g., healthy take-away options or convenience foods), dietary patterns and meal-timings, dietary support and/or environment and other practical recommendations may be beneficial to support these changes [20]. 

### 4.4. Strengths and Limitations

This systematic review found that tactical personnel in general exceeded protein recommendations. The studies also reported inadequate energy and carbohydrate intake and excessive fat intake of tactical participants in comparison to the MDRI. While included studies reflected the usual limitations of applied dietary research, (e.g., small sample sizes, heterogeneity of data, and the existence of under-reporting), the systematic review has pooled all available data to synthesize the best-available evidence. Due to the heterogeneity of studies, no meta-analysis or GRADE analysis were able to be conducted. While there was some representation across military and law enforcement personnel, there were limited studies across other tactical occupations (e.g., fire and rescue).

Interestingly, while this systematic review has provided some useful insights into the dietary intake of tactical personnel that can inform dietary interventions, it has not provided insight into the dietary patterns and meal timings of tactical personnel. These were largely unreported in the included studies; in part due to the common use of a food frequency questionnaire (FFQ) to estimate dietary intake in the included studies. This tool is useful in tactical occupations as they often do not readily support ease of recording through other methods (e.g., food or photo diary point-in-time assessments). While diet recall was used by several studies [42,54,57,58,61], and does not have this limitation, it is limited by recall bias [89]. High stress or unstructured environments or high use of convenience or takeaway foods may not support accurate recall or dietary timings. Like all studies that incorporate dietary assessment, under-reporting should be acknowledged and considered particularly when interpreting data that suggests under-consumption. 

As they are designed for military personnel, future studies could inform an update of the current MDRI guidelines and, whether they are suitable to assess the dietary intake for all tactical personnel. In addition, guidelines that consider performance optimization, and therefore relevant sports and performance guidelines, may also be beneficial [18,31,64,90]. Large scale studies are also needed to identify the healthy eating barriers as this population face specific occupation-related barriers, such as shift work and working under extreme conditions [20]. Similarly, exploring RED-S or the long-term health effects of military occupations which incorporate phases of energy deficiency and recovery, characteristic of tactical occupations, is warranted [69]. 

Within this systematic review, sex was reported to varying levels and the authors did not receive any reply for raw data of the sex from the three papers with unspecified sex distributions. One publication was transcribed using Google Translate^®^ and we acknowledge that extraction via this method is more prone to error than extraction of English language articles [38]. 

## 5. Conclusions

Based on the available evidence, tactical personnel, in general, met dietary protein and exceeded dietary fat recommendations but failed to meet energy and carbohydrate recommendations. Therefore, practical, and individualized approaches to support (1) an adequate, dietary energy and carbohydrate intake; (2) controlled dietary fat intake with consideration of population recommendations of reducing saturated fat and prioritizing unsaturated fat intake; and (3) moderate, high-quality protein intake that is not prioritized at the expense of other macronutrients; are warranted. 

Further research is needed to investigate nutrition interventions, dietary patterns, and barriers to dietary intake in tactical personnel. In addition, further consideration of updating national references guidelines for these occupations is warranted. 

## Figures and Tables

**Table 1:** Eligibility criteria for each domain for a systematic review of studies reporting dietary intake in tactical personnel, according to the Preferred Reporting Items for Systematic Reviews and Meta-Analyses framework [32].

Domain	Inclusion Criteria	Exclusion Criteria
Population	Tactical personnel (including military personnel, law enforcement personnel, fire and rescue personnel, and active-services personnel), ≥18 years, that are active-services personnel (full time or part time)	Tactical personnel with medical conditions or medications that influence metabolism or nutrition requirements, pregnant women, and disabled active services personnel
Intervention	Participants following a free-living diet with no restrictions or food provided	Dietary supplementation Diets for special training or altitude Food provided to participants
Comparison	No comparison groups required	
Outcomes	The primary outcomes include dietary intake of energy, carbohydrate, protein and/or fat	No outcomes of interest reported.
Study design	Original research published from 1990 up until 28 October 2020	Conference papers, dissertations, abstracts without full text, and protocol papers where we were unable to identify a published result paper.

**Table 2:** Study and sample characteristics of included studies (N = 22; Mean ± SD).

Author, year	Location	Sex	Sample	Age (years)	Height (cm)	Weight (kg)	BMI (kg/m^2^)
Military personnel (n = 16)							
Copp et al. [42], 1991	USA	Male	30	35 ± 6	182 ± 6	82 ± 8	25 ^2^
Hart et al. [43], 1992	USA	NR ^1^	118	31 ± 5	180 ± 6	79 ± 8	24 ^2^
Deuster et al. [44], 2003	USA	NR	38	25 (SE1)	178 (SE 1)	82 (SE 1)	26 ^2^
Viravathana et al. [45], 2005	Thailand	Male	108	37 ± 7	168 ± 6	64 ± 7	23 ± 2
Israeli et al. [46], 2008	Israel	Female	216	19 ± 1	162 ± 6	60 ± 10	23 ± 3
		Male	78	19 ± 1	174 ± 7	70 ± 13	23 ± 4
Etzion-Daniel et al. [47], 2008	Israel	Female	92	NR	163 ± 6	61 ± 10	23 ± 3
		Male	33	NR	176 ± 7	67 ± 9	22 ± 3
Mullie et al. [48], 2009	Belgium	Male	95	43 ± 7	NR	NR	NR
Nkondjock et al. [49], 2010 ^3^	Cameroon	Female	60	37 ± 10	NR	NR	NR ^3^
		Male	473		NR	NR	NR ^3^
Margolis et al. [50], 2012	USA	Female	91	23 ± 6	163 ± 6	66 ± 8	25 ± 3
		Male	118	23 ± 5	176 ± 7	84 ± 16	27 ± 4
Carlson et al. [51], 2013	USA	Female	7	23 ± 3	173 ± 11	73 ± 12	24 ± 2
		Male	12				
Ramsey et al. [52], 2013	USA	Female	21	36 ± 12	164 ± 5	65 ± 13	24 ± 4
		Male	18	36 ± 10	174 ± 7	90 ± 14	30 ± 3
Fallowfield et al. [53], 2014	UK	NR	202	28 ± 7	179 ± 0.1	83 ± 9	26 ± 2
Beals et al. [54], 2015	USA	Female	55	27 ± 6	164 ± 8	65 ± 10	24 ± 3
		Male	269	28 ± 7	177 ± 10	84 ± 12	27 ± 4
Clemente et al. [55], 2015	Italy	Male	106	21 ± 2	NR	NR	24 ± 3
Rahmani et al. [56], 2018 ^4^	Iran	Male	246	24 ± 2	NR	77 ± 15	24 ± 4
Stark et al. [57], 2019	Israel	Male	31	25 (SE 1)	179 (SE 0.01)	74 (SE 2)	23 (SE 0.4)
Law enforcement personnel (n = 4)							
Briley et al. [26], 1990	USA	Female	11	35 ± 6	NR	NR	NR
		Male	13	38 ± 8	NR	NR	NR
Donadussi et al. [58], 2009	Brazil	Male	183	35 ± 6	NR	NR	27 ± 4
Gibson et al. [59], 2017	UK	Female	1510	40 ± 10	NR	NR	26 ± 5
		Male	2568	42 ± 9	NR	NR	28 ± 4
Kosmadopoulos et al. [60], 2020	Canada	Female	6	32 ± 4	NR	NR	24 ± 2
		Male	25	32 ± 6	NR	NR	25 ± 2
Firefighters (n = 2)							
Bonnell et al. [61], 2017	Australia	Female	1	36 (29–51) ^5^	NR	NR	25 (23–27) ^5^
		Male	18		NR	NR	
Johnson et al. [62], 2020	USA	Male	150	37 ± 8	NR	NR	28 ± 4

Abbreviations: BMI, body mass index; SE, standard error; USA, United States of America; UK, United Kingdom; ^1^ NR = not reported; not reported in the manuscript; ^2^ BMI was calculated from reported height and weight values. The formula is BMI = kg/m^2^ where kg is a person’s weight in kilograms and m^2^ is their height in meters squared; ^3^ Nkondjock et al. [49], reported BMI across three strata however, the sample size per each strata is not reported to combine means and standard deviations; ^4^ Rahmani et al. [56], stratified participants into four groups based on Quartiles of AHEI-2010. Combined groups of means and Standard deviations into a single group by Cochrane’s formula; ^5^ Bonnell et al. [61], reported age and body mass index as median (IQR).

**Table 3:** Reported energy and macronutrient intakes of female and male tactical personnel in the included studies (N = 22).

Author, Year	Sex	Energy kcal/day	Compared to MDRI: F = 2300 kcal/d M = 3250 kcal/d	Protein g/day	Compared to MDRI: F = 72 g/day M= 91 g/day	Carbohydrate % of Energy	Compared to MDRI: ≥55% Total Energy	Fat % of Energy	Compared to MDRI: ≤30% Total Energy
Military personnel (n = 16)									
Copp et al. [42], 1991	Male	2585 ± 776	80% of MDRI	103 ^2^	113% of MDRI	48 ± 9	Below MDRI	34 ± 10	Above MDRI
Hart et al. [43], 1992	NR ^1^	2729 ± 803	-	102 ^2^	-	45 ± 8	Below MDRI	34 ± 6	Above MDRI
Deuster et al. [44], 2003	NR	2962 (SE 239)	-	135 (SE 14)	-	41 ^2^	Below MDRI	38 ^2^	Above MDRI
Viravathana et al. [45], 2005	Male	2304 ± 645	71% of MDRI	81 ± 25	89% of MDRI	53 ± 9	Below MDRI	30 ± 7	Meeting MDRI
Israeli et al. [46], 2008	Female	2210 ± 946	96% of MDRI	82 ± 38	114% of MDRI	53 ^2^	Below MDRI	33 ^2^	Above MDRI
	Male	2656 ± 1068	82% of MDRI	106 ± 47	116% of MDRI	50 ^2^	Below MDRI	33 ^2^	Above MDRI
Etzion-Daniel et al. [47], 2008	Female	1993 ± 736	87% of MDRI	87 ± 34	121% of MDRI	53 ^2^	Below MDRI	34 ^2^	Above MDRI
	Male	2368 ± 723	73% of MDRI	88 ± 31	97% of MDRI	52 ^2^	Below MDRI	34 ^2^	Above MDRI
Mullie et al. [48], 2009 ^3^	Male	3100 ± 1079	95% of MDRI	119 ± 37	131% of MDRI	42 ± 7	Below MDRI	37 ± 8	Above MDRI
Nkondjock et al. [49], 2010 ^4^	Female	1852 ± 1356	81% of MDRI	NR	-	NR	-	NR	-
	Male	2052 ± 1356	63% of MDRI	NR	-	NR	-	NR	-
Margolis et al. [50], 2012	Female	1824 ± 1014	79% of MDRI	69 ± 38	96% of MDRI	49 ^2^	Below MDRI	36 ^2^	Above MDRI
	Male	1975 ± 909	61% of MDRI	78 ± 36	86% of MDRI	49 ^2^	Below MDRI	35 ^2^	Above MDRI
Carlson et al. [51], 2013	NR ^5^	3231 ± 2215	-	117 ± 90	-	51 ^2^	Below MDRI	33 ^2^	Above MDRI
Ramsey et al. [52], 2013	Female	1975 ± 639	86% of MDRI	87 ± 32	121% of MDRI	54 ^2^	Below MDRI	31 ^2^	Above MDRI
	Male	2639 ± 1252	81% of MDRI	100 ± 51	110% of MDRI	44 ^2^	Below MDRI	33 ^2^	Above MDRI
Fallowfield et al. [20], 2014	NR	3173 + 854 ^2^	-	127 ^2^	-	46 ± 7	Below MDRI	35 ± 6	Above MDRI
Beals et al. [54], 2015	Female	1920 ± 956	83% of MDRI	84 ± 47	116% of MDRI	53 ^2^	Below MDRI	29 ± 9	Meeting MDRI
	Male	2574 ± 974	79% of MDRI	116 ± 56	127% of MDRI	49 ^2^	Below MDRI	32 ± 10	Above MDRI
Clemente et al. [55], 2015 ^6^	Male	3071 ± 737	94% of MDRI	120 ± 30	132% of MDRI	56 ^2^	Meeting MDRI	28 ^2^	Meeting MDRI
Rahmani et al. [56], 2018 ^7^	Male	2232 ± 504	69% of MDRI	104 ± 21	114% of MDRI	49 ^2^	Below MDRI	30 ^2^	Meeting MDRI
Stark et al. [57], 2019	Male	2657 (SE 168)	82% of MDRI	113 (SE 9)	124% of MDRI	47 ^2^	Below MDRI	36 ^2^	Above MDRI
Law enforcement personnel (n = 4)									
Briley et al. [26], 1990	NR ^8^	2273 ± 694	-	91 ^2^	-	41 ± 7	Below MDRI	41 ± 6	Above MDRI
Donadussi et al. [58], 2009	Male	2230 ± 812	69% of MDRI	NR	-	NR	-	39 ± 8	Above MDRI
Gibson et al. [59], 2017	Female	1711 ± 395	74% of MDRI	71 ^2^	99% of MDRI	45 ± 6	Below MDRI	31 ± 6	Above MDRI
	Male	2107 ± 502	65% of MDRI	90 ^2^	99% of MDRI	44 ± 6	Below MDRI	34 ± 5	Above MDRI
Kosmadopoulos et al. [60], 2020 ^9^	NR ^10^	159 + 52 ^11^	-	28 ± 10 ^11^	-	70 ± 27 ^10^	Meeting MDRI	58 ± 25 ^10^	Above MDRI
Firefighters (n = 2)									
Bonnell et al. [61], 2017 ^12^	-	-	-	-	-	-	-	-	-
Johnson et al. [62], 2020	Male	2292 ± 630	71% of MDRI	123 ± 45	135% of MDR	37 ± 10	Below MDRI	39 ± 9	Above MDRI

Abbreviations: MDRI, Military Dietary Reference Intakes; ^1^ NR = Not reported; either not reported in the manuscript. Note: if a study did not report sex the total energy and protein intake could not be compared to the MDRI; ^2^ Data was calculated by converting kilojoules to kcal and/or converting grams or kcal to % energy intake; ^3^ Mullie et al. [48], reported data across three different dietary assessment methodologies (n = 95 each). Combined groups of means and Standard deviations into a single group by Cochrane’s formula; ^4^ Nkondjock et al. [49], reported data across three different personnel including officers, warrant officers, and enlisted men. Combined groups of means and standard deviations into a single group by Cochrane’s formula. Participant numbers for each group were obtained from the sex variable; ^5^ Carlson et al. [51], reported the number of males and females in baseline characteristics however, did not separate dietary intake per sex; ^6^ Clemente et al. [55], stratified participants into two groups based on Intimal Media Thickness of <0.7 and ≥0.7 mm. Combined groups of means and standard deviations into a single group by Cochrane’s formula; ^7^ Rahmani et al. [56], stratified participants into four groups based on Quartiles of AHEI-2010. Combined groups of means and standard deviations into a single group by Cochrane’s formula; ^8^ Briley et al. [26], reported the number of males and females in baseline characteristics however, did not separate dietary intake per sex; ^9^ Kosmadopoulos et al. [60], stratified participants into four groups based on different types of shifts. Combined groups of means and standard deviations into a single group by Cochrane’s formula; ^10^ Kosmadopoulos et al. [60], reported the number of males and females in baseline characteristics; however, did not separate dietary intake per sex; ^11^ Kosmadopoulos et al. [60], reported total overall and macronutrient caloric intake expressed as percentages of BMR; ^12^ Bonnell et al. [61], stratified participants into four groups based on different types of shifts. All values were reported ad median (IQR) hence unable to combine groups by Cochrane’s formula.

## Data Availability

Data can be requested from the corresponding author.

## Supplementary Materials

The following are available online at https://www.mdpi.com/article/10.3390/nu13103502/s1, Table S1: Search strategy implemented across four electronic databases from 1990 up until 28 October 2020. Table S2: Quality assessment as per the Academy of Nutrition and Dietetics Quality Criteria Checklist.
