# Supplementary material for: Free-Living Dietary Intake in Tactical Personnel and Implications for Nutrition Practice: A Systematic Review
Source: Nutrients. 2021 Oct 3;13(10):3502. doi: 10.3390/nu13103502 (PMC8537156; doi:10.3390/nu13103502)
Supplement: Supplementary file 1 [file nutrients-13-03502-s001.zip › nutrients-1394308-supplementary.pdf]

Systematic Review

Free-Living Dietary Intake in Tactical Personnel and Implications for Nutrition Practice: A Systematic Review

Kristen L MacKenzie-Shalders[1*], Angela Tsoi[1], Ka Wing Lee[1], Charlene Wright[1,2], Gregory Cox[1], and Robin Orr[3]

[1] Nutrition and Dietetics, Faculty of Health Sciences and Medicine, Bond University, Queensland: Australia, 4226; kmackenz@bond.edu.au; angela.tsoi@student.bond.edu.au; kawing.lee@student.bond.edu.au; gcox@bond.edu.au; cwright@bond.edu.au
[2] School of Medicine and Dentistry, Centre of Applied Health Economics and Menzies Health Institute Queensland, Griffith University; charlene.wright@griffithuni.edu.au
[3] Tactical Research Unit, Bond University, Queensland: Australia, 4226
* Correspondence: kmackenz@bond.edu.au; Tel.: +61755951018

SUPPLEMENTARY MATERIAL

Table S1: Search strategy implemented across four electronic databases from 1990 up until 28 October 2020
Table S2: Quality assessment as per the Academy of Nutrition and Dietetics Quality Criteria Checklist.

Table S1: Search strategy implemented across four electronic databases from 1990 up until 28 October 2020

MEDLINE (via PubMed) - searched 28 October 2020 using keywords (title and abstract) and MeSH Terms. Result = 4,841 records
(“Police”[MeSH Terms] OR “law enforcement”[MeSH Terms] OR “Emergency Responders”[MeSH Terms] OR "military health"[MeSH Terms] OR “military personnel”[MeSH Terms] OR “Firefighters”[MeSH Terms] OR "tactical personnel"[Title/Abstract] OR "Emergency worker"[Title/Abstract] OR Military[Title/Abstract] OR army[Title/Abstract] OR "Customs Officer"[Title/Abstract] OR Marines[Title/Abstract] OR "Defence personnel"[Title/Abstract] OR "National guard"[Title/Abstract] OR "national guards"[Title/Abstract] OR FBI[Title/Abstract] OR sheriff*[Title/Abstract] OR "Patrol officer"[Title/Abstract] OR Firefighter*[Title/Abstract] OR Fireman[Title/Abstract] OR firemen[Title/Abstract] OR "Law Enforcement"[Title/Abstract] OR Police*[Title/Abstract] OR "Emergency Responder"[Title/Abstract] OR "emergency responders"[Title/Abstract] OR SES[Title/Abstract] OR "state emergency service"[Title/Abstract] OR "fire rescue"[Title/Abstract])<br>AND<br>(“Diet”[MeSH Terms] OR “Nutrients”[MeSH Terms] OR “Feeding Behavior”[MeSH Terms] OR “eating”[MeSH Terms] OR “Dietary Carbohydrates”[MeSH Terms] OR “Nutrition assessment”[MeSH Terms] OR “Diet Surveys”[MeSH Terms] OR “Diet Records”[MeSH Terms] OR Diet*[Title/Abstract] OR Nutr*[Title/Abstract] OR "food intake"[Title/Abstract] OR "Eating behaviour"[Title/Abstract] OR "eating behaviours"[Title/Abstract] OR "eating patterns"[Title/Abstract] OR "eating pattern"[Title/Abstract] OR "eating habit"[Title/Abstract] OR "eating habits"[Title/Abstract] OR "food recall"[Title/Abstract] OR "food recalls"[Title/Abstract] OR "food record"[Title/Abstract] OR Macronutrient*[Title/Abstract] OR Carbohydrat*[Title/Abstract] OR Fat[Title/Abstract])
CINAHL (via Ebscohost) was searched on 28 October 2020 using keywords and CINAHL Headings. Results 4080 records
((MH "Police") OR (MH "Military Health") OR (MH "Military Personnel+") OR (MH "Firefighters") OR (TI "tactical personnel" OR AB "tactical personnel") OR (TI "Emergency worker" OR AB "Emergency worker") OR (TI Military OR AB Military) OR (TI army OR AB army) OR (TI "Customs Officer" OR AB "Customs Officer") OR (TI Marines OR AB Marines) OR (TI "Defence personnel" OR AB "Defence personnel") OR (TI "National guard" OR AB "National guard") OR (TI "national guards" OR AB "national guards") OR (TI FBI OR AB FBI) OR (TI sheriff* OR AB sheriff*) OR (TI "Patrol officer" OR AB "Patrol officer") OR (TI Firefighter* OR AB Firefighter*) OR (TI Fireman OR AB Fireman) OR (TI firemen OR AB firemen) OR (TI "Law Enforcement" OR AB "Law Enforcement") OR (TI Police* OR AB Police*) OR (TI "Emergency Responder" OR AB "Emergency Responder") OR (TI "emergency responders" OR AB "emergency responders") OR (TI SES OR AB SES) OR (TI "state emergency service" OR AB "state emergency service") OR (TI "fire rescue" OR AB "fire rescue"))<br>AND<br>((MH "Diet+") OR (MH "Nutrients+") OR (MH " Eating Behavior+") OR (MH "Eating") OR (MH "Dietary Carbohydrates+") OR (MH "Nutrition assessment") OR (MH "Diet Records") OR (TI Diet* OR AB Diet*) OR (TI Nutr* OR AB Nutr*) OR (TI "food intake" OR AB "food intake") OR (TI "Eating behaviour" OR AB "Eating behaviour") OR (TI "eating behaviours" OR AB "eating behaviours") OR (TI "eating patterns" OR AB "eating patterns") OR (TI "eating pattern" OR AB "eating pattern") OR (TI "eating habit" OR AB "eating habit") OR (TI "food recall" OR AB "food recall") OR (TI "food recalls" OR AB "food recalls") OR (TI "food record" OR AB "food record") OR (TI Macronutrient* OR AB Macronutrient*) OR (TI Carbohydrat* OR AB Carbohydrat*) OR (TI Fat* OR AB Fat*))
Embase (via Ovid) was searched on 28 October 2020 using keywords and MeSH Headings. Results = 6355 records

'police'/exp OR 'law enforcement'/exp OR 'rescue personnel'/exp OR 'military health'/exp OR 'military personnel'/exp OR 'fire fighter'/exp OR 'tactical personnel':ti,ab OR 'emergency worker':ti,ab OR military:ti,ab OR army:ti,ab OR 'customs officer':ti,ab OR marines:ti,ab OR 'defence personnel':ti,ab OR 'national guard':ti,ab OR 'national guards':ti,ab OR fbi:ti,ab OR sheriff*:ti,ab OR 'patrol officer':ti,ab OR firefighter*:ti,ab OR fireman:ti,ab OR firemen:ti,ab OR 'law enforcement':ti,ab OR police*:ti,ab OR 'emergency responder':ti,ab OR 'emergency responders':ti,ab OR ses:ti,ab OR 'state emergency service':ti,ab OR 'fire rescue':ti,ab<br>AND<br>'Diet'/exp OR 'Nutrients'/exp OR 'feeding behavior'/exp OR 'eating'/exp OR 'eating habit'/exp OR 'carbohydrate intake'/exp OR 'nutritional assessment'/exp OR 'diet'/exp OR 'medical record'/exp OR diet*:ti,ab OR nutr*:ti,ab OR 'food intake':ti,ab OR 'eating behaviour':ti,ab OR 'eating behaviours':ti,ab OR 'eating patterns':ti,ab OR 'eating pattern':ti,ab OR 'eating habit':ti,ab OR 'eating habits':ti,ab OR 'food recall':ti,ab OR 'food recalls':ti,ab OR 'food record':ti,ab OR macronutrient*:ti,ab OR carbohydrat*:ti,ab	
Web of Science was searched 28 October 2020 for the following keywords in topic or title (limits:). Results = 3302 records	
(TI=(Diet* OR Nutr* OR "food intake" OR "Eating behaviour" OR "eating pattern" OR "eating habit" OR "food recall" OR "food recalls" OR "food record" OR Macronutrient*) OR AB=(Diet* OR Nutr* OR "food intake" OR "Eating behaviour" OR "eating pattern" OR "eating habit" OR "food recall" OR "food recalls" OR "food record" OR Macronutrient*)) AND (TI=("tactical personnel" OR "Emergency worker" OR Military OR army OR "Customs Officer" OR "Defence personnel" OR "National guard" OR FBI OR sheriff* OR "Patrol officer" OR Firefighter* OR Fireman OR firemen OR "Law Enforcement" OR Police* OR "Emergency Responder" OR "emergency responders" OR SES OR "state emergency service" OR "fire rescue") OR AB=("tactical personnel" OR "Emergency worker" OR Military OR army OR "Customs Officer" OR "Defence personnel" OR "National guard" OR "national guards" OR FBI OR sheriff* OR "Patrol officer" OR Firefighter* OR Fireman OR firemen OR "Law Enforcement" OR Police* OR "Emergency Responder" OR "emergency responders" OR SES OR "state emergency service" OR "fire rescue"))	
Total	*18578 records*

Table S2: Quality assessment as per the Academy of Nutrition and Dietetics Quality Criteria Checklist.

Author, year	1. Was the research question clearly stated?	2. Was the selection of study subjects free from bias?	3. Were study groups comparable?	4. Was method of handling withdrawals described?	5. Was blinding used to prevent introduction of bias?	6. Were the intervention and any comparisons described in detail? Were intervening factors described?	7. Were outcomes clearly defined and the measurements valid and reliable?	8. Was the statistical analysis appropriate for the study design and type of outcome indicators?	9. Are conclusions supported by results with biases and limitations taken into consideration?	10. Is bias due to study's funding or sponsorship unlikely?	Overall study quality
Beals et al., 2015	Yes	Yes	Yes	No	N/A	No	Yes	Yes	Yes	No	Positive
Bonnell et al., 2017	Yes	Yes	N/A	No	N/A	N/A	Yes	Yes	Yes	N/A	Positive
Briley et al., 1990	Yes	No	Yes	Yes	N/A	N/A	Yes	Yes	Yes	N/A	Positive
Carlson et al., 2013	Yes	Yes	Yes	N/A	N/A	N/A	Yes	Yes	Yes	N/A	Positive
Clemente et al., 2015	Yes	Yes	Yes	No	N/A	N/A	Yes	Yes	Yes	N/A	Positive
Copp et al., 1991	Yes	No	N/A	No	N/A	Yes	Yes	Yes	No	No	Positive
Deuster et al., 2003	Yes	No	N/A	No	N/A	Yes	Yes	Yes	No	No	Positive
Donadussi et al., 2009	Yes	Yes	N/A	No	N/A	N/A	Yes	Yes	No	N/A	Neutral
Etzion-Daniel et al., 2008	Yes	No	N/A	No	N/A	N/A	Yes	Yes	Yes	N/A	Neutral
Fallowfield et al., 2014	Yes	No	N/A	Yes	N/A	Yes	Yes	Yes	Yes	No	Positive
Gibson et al., 2017	Yes	Neutral	N/A	No	N/A	Yes	Yes	Yes	Yes	No	Positive
Hart et al., 1992	Yes	Yes	No	No	N/A	N/A	Yes	Yes	No	No	Positive
Israeli et al., 2008	Yes	Yes	Yes	No	N/A	N/A	Yes	Yes	No	N/A	Positive
Johnson et al., 2020	Yes	Yes	N/A	Yes	N/A	N/A	Yes	Yes	Yes	N/A	Positive
Kosmadopoulos et al., 2020	Yes	No	N/A	Yes	N/A	No	No	Neutral	Yes	Yes	Neutral
Margolis et al., 2012	Yes	Yes	N/A	No	N/A	Yes	Yes	Yes	Yes	Yes	Positive

Mullie et al., 2009	Yes	Yes	No	No	N/A	N/A	Yes	Yes	No	N/A	Neutral
Nkondjock et al., 2010	Yes	No	N/A	No	N/A	No	Yes	Yes	Yes	No	Positive
Rahmani et al., 2018	Yes	Yes	Yes	No	N/A	N/A	Yes	Neutral	Yes	No	Positive
Ramsey et al., 2013	Yes	Yes	N/A	No	N/A	N/A	Yes	Yes	Yes	N/A	Positive
Stark et al., 2019	Yes	Yes	N/A	No	N/A	N/A	Yes	Yes	No	No	Neutral
Viravathana et al., 2005	Yes	Yes	N/A	No	N/A	No	Yes	Yes	No	N/A	Neutral
